# Comprehensive sexuality education in Hong Kong: study protocol for process and outcome evaluation

**DOI:** 10.1186/s12889-021-10253-6

**Published:** 2021-01-22

**Authors:** Ellie Bostwick Andres, Edmond Pui Hang Choi, Alice Wai Chi Fung, Kevin Wing Chung Lau, Neda Hei Tung Ng, Monique Yeung, Janice Mary Johnston

**Affiliations:** 1 School of Public Health, University of Hong Kong, Patrick Manson Building, (North Wing), 7 Sassoon Road, Pokfulam, Hong Kong; 2 School of Nursing, University of Hong Kong, 4/F, William M.W. Mong Block, 21 Sassoon Road, Pokfulam, Hong Kong; 3 Mother’s Choice, 10 Borrett Road, Mid-Levels, Hong Kong

**Keywords:** Sex education, Sexual health, Adolescent behaviour, Health promoting school

## Abstract

**Background:**

Hong Kong lacks comprehensive school-based sexuality education. Recent public health concerns have brought the inadequacies of sex education in Hong Kong to the forefront. The aim of the proposed study is to develop and evaluate the effectiveness of a comprehensive school-based sexuality education program in Hong Kong.

**Methods:**

The proposed study is a prospective longitudinal study implemented in six secondary schools in Hong Kong over two academic years. The study adopts an ecological approach providing informational workshops for students, teachers and school management, social workers and guidance counsellors and parents. Study outcomes will be evaluated through pre- and post-tests.

**Results:**

Key outcomes of interest among students include sexual health knowledge, awareness of values motivating healthy sexual decisions, understanding and efficacy of sexual communication and intention to use contraception. Among school employees and parents key outcomes include self-efficacy to engage in sexual health discussions with students/children, sexual health knowledge and awareness of Hong Kong community sexual health resources.

**Conclusions:**

The proposed study will result in the development of a tested school-based culturally relevant comprehensive sexual health education program. Ultimately, this program aims to not only empower adolescents and their trusted adults in building a supportive environment for sexual health promotion but also construct a learning network to generate longitudinal evidence for the effectiveness of comprehensive sexuality education in improving sexual health outcomes. The program has the potential for expansion through widespread adoption in Hong Kong schools to benefit more adolescents and reduce the medical and societal burdens related to crisis pregnancy, sexually transmitted infections and sexual abuse.

## Background

Hong Kong is considered ‘Asia’s world city’ and the most westernized Chinese society due to its heritage as a former British colony. However, in contrast to its peer international cities, sexuality is not as openly discussed or expressed in Hong Kong and remains somewhat taboo [1]. Hong Kong adolescents generally have a higher age at first sex and fewer sexual experiences than in western contexts. The most recent Youth Sexuality Study (conducted every 5 years by the Family Planning Association of Hong Kong) found 6 and 7% of Hong Kong high school (Grades 10–12) female and male students, respectively, reported a sexual intercourse experience, whereas a comparative study of 15-year-olds from five western countries (the United States, Scotland, Finland, France and Poland) found a prevalence of sexual initiation ranging from 12 to 37% [2, 3].

Hong Kong’s relatively low levels of youth sexual activity are not indicative of substantial or effective sexual education. The Education Bureau first encouraged sex education in school as early as 1971, however, it has never been integrated as a part of the formal curriculum, but rather relegated to special assemblies or function days left to individual schools’ discretion [4, 5]. As Che concludes in her examination of sex education in Hong Kong schools, “the importance of sex education has always been subsidiary as it is not an examination subject.” [5] Individual schools have the flexibility to tailor the sexual education approach, content and delivery mode in accordance with their background, mission, ethos and resources [4].

Recent public health concerns have brought the inadequacies of sexual education in Hong Kong to the forefront. Sexually transmitted infections (STIs) are not notifiable locally in Hong Kong and no population-based STI prevalence studies are regularly conducted; hence, STI data is mostly anecdotal or limited to certain key populations or settings [6, 7]. However, the first population-based study of chlamydia trachomatis among Hong Kong residents conducted in 2016 found a 5.5 and 4.8% prevalence among sexually active females and males aged 18–26, respectively, higher rates than the United Kingdom, United States and China [7–10]. Moreover, HIV infections reported by the Department of Health surged from 181 cases in 1997 to 624 in 2018, with 30% of cases diagnosed among adults aged 20–29 [11].

Meanwhile, Hong Kong adolescents’ knowledge and understanding related to sexual health is insufficient and appears to be declining. The Family Planning Association of Hong Kong’s 2016 Youth Sexuality Study found high school aged students (Forms 3–6) scored an average of 67% correct on questions about conception, STIs and HIV/AIDS in 2016, down from 75% in 2011 [12]. Middle school students (Forms 1–2), already unsatisfactory with only 50% correct on average in 2011, dropped to 42% in 2016 [12]. Both knowledge and practice related to contraceptive use appears particularly deficient, with less than half of sexually active youth reporting contraceptive use every time [13].

Moreover, while the magnitude of teenage pregnancies has not been adequately explored in Hong Kong, an estimated 4700 females aged 15 to 24 faced crisis pregnancies in 2016^1^ [14, 15] 2 [16]. Crisis pregnancies are yet another symptom of inadequate education and support systems to prepare adolescents for the demands of adulthood. Furthermore, a recent survey of Hong Kong high school students attests to a distressing trend in sexual harassment and sexting. Thirteen percent of student respondents admitted to sexually harassing others, while 7.8% reported they had personally been sexually harassed [17]. More than a quarter of respondents indicated receipt of sexting messages. The local data stress the urgent need to enhance sexual health for Hong Kong adolescents.

Hong Kong adolescents are ill-equipped to make informed decisions about sexual health. The above evidence suggests schools’ autonomy implementing adolescent sexual education may compromise its efficacy. Due to a lack of holistic comprehensive sexual health education in Hong Kong, adolescents facing challenging situations requiring knowledge, support and inclusion are often forced to make isolated and uninformed decisions, which may lead to high-risk behaviours, such as sex at an early age, unprotected sex or a combination of sex and substance abuse. There is an urgent need for a holistic, replicable and sustainable sexuality education model.

### Intervention

Based on 30 years of experience working with adolescents facing crisis pregnancies in Hong Kong, Mother’s Choice, a local Hong Kong charity, developed a comprehensive sexual health education program, *Sexuality 360 @ School,* to meet Hong Kong adolescents’ unique needs. In contrast to the pervasive understanding in Hong Kong culture that talking about sex promotes sex among adolescents, the program seeks to build on international evidence indicating increased dialogue between adolescents and trusted adults about sexuality serves as a protective factor delaying sexual activity [18, 19].

The program seeks to develop an ecosystem that empowers adolescents and key adults in their support networks (i.e. school and family) with sexual health knowledge, attitudes, skills and connection to motivate healthy sexual decision-making. The program applies the ecological perspective to target four key groups within the school community who influence adolescents’ sexual behaviour: secondary school students (Form 1 – Form 4), school management and teachers, school social workers and guidance counsellors, and parents. The age range of students is approximately 12 to 16 years. The program recognizes the importance of parents and teachers in creating a comprehensive sexual health program for adolescents [20]. The program is particularly cognizant of engaging parents within a culture where parents may not have identified their own values towards sexuality, much less understood how to cope with or respond to their children’s sexuality concerns. Likewise, teachers, responsible for delivering sexual health information have indicated insufficient resources or support, time and/or skills to implement sexuality education [4].

### Theoretical and conceptual approaches

*Sexuality 360* draws from several key evidence-based programs and theoretical approaches to fashion a comprehensive sexual health education program uniquely suited for Hong Kong youth. Since sexual health is a product of a myriad of interconnected physical, social and psychological factors, it requires a holistic approach in the context in which these factors interact. School provides such a context as the fundamental setting in which adolescents develop their gender identities, norms, values and sense of self – key determinants of sexual well-being. The learning process extends far beyond the formal curriculum into everyday practices and interactions with peers and teachers in school.

### Health promoting school

*Sexuality 360* adapts the World Health Organization’s Health Promoting School (HPS) approach [21]. HPS aims to provide a multifaceted response to the health needs of students by strengthening schools’ capacity as healthy settings for living, learning and working, engaging school management, teachers, students, parents, health providers and community leaders. HPS takes a holistic approach to influence health-related knowledge, beliefs, skills, attitudes, and values. Systematic reviews of HPS programs in high-income countries show the approach has helped reduce important risk factors, such as BMI [22] and tobacco use [23]. The WHO recommends HPS as an effective strategy for HIV/AIDS/STI prevention. *Sexuality 360 @ School* applies the HPS model to sexual health for a strategic, cost-effective and sustainable approach to reducing sexual health risks among adolescents in Hong Kong. *Sexuality 360 @ School* focuses on three key components of HPS: engaging cross-sectorial sexual health professionals in the community, providing skills-based sexuality education to adolescents and trusted adults in schools and improving sexual health promoting policy in school.

### School-based sexuality education

Schools are considered ideal settings for implementing comprehensive sexuality education since school authorities can regulate many aspects of the learning environment to make it supportive and protective [24]. The school setting also provides an environment where sexuality education can be delivered in developmentally relevant sequence over several grades, building knowledge successively [25]. Research also shows comprehensive sexuality education, when delivered appropriately, empowers adolescents to protect themselves physically, mentally, and emotionally [26]. It encourages abstinence, delays sexual initiation, reduces the number of sexual partners, and increases consistent contraceptive use if adolescents are already sexually active [24]. Studies have shown multiple conversations with adolescents on the topic of sexuality, over a period of time, are crucial to the success of a sexuality education program [24].

### Parental involvement in school-based sexuality education program

The *ETR Get Real: Comprehensive Sexuality Education That Works* program is an evidence-based program emphasizing social and emotional skills as a key component of healthy relationships and responsible decision making [27]. It uniquely supports parents as the primary sexuality educators for their children through family activities encouraging dialogue with students about sexual health. *Get Real* has been shown to delay sex among middle school participants, with 16% fewer boys and 15% fewer girls initiating sex by the end of eighth grade compared to students receiving sexuality education “as usual” in comparison schools [27]. Drawing from *Get Real*, *Sexuality 360 @ School* includes parent training and regular parent updates on the sexuality education children receive in school.

### Sexuality 360@school –student intervention

The student curriculum consists of 12 workshops, grouped into 3 topics per grade. Each workshop is 1 h and designed for a class of 25 students or fewer to maximize interaction between educators and students. The full curriculum is designed to be delivered across 4 years, beginning in Form 1 and building through Form 4. The curriculum is suitable for all adolescents, regardless of their sexual experience or orientation.

The goal of the workshops is to directly equip students with knowledge and skills needed to make healthy sexual decisions. The approach is comprehensive, age-appropriate and sex-positive in nature addressing broad aspects of sexuality including human development, relationships, decision-making, communication, consent, contraception, unintended pregnancy, and disease prevention. The structure is organized in a progressive, coordinated manner in which issues are revisited with increasing depth as students mature. In terms of teaching strategy, interactivity and learner-centric are core principles. In addition to face-to-face interaction with students, we have developed four short videos to complement our workshop teaching materials. Given sexuality is a difficult and sensitive topic, these videos can also serve as tools to equip teachers in facilitating sexuality education workshops.

### Sexuality 360@school – teacher and management intervention

The teacher and management workshops aim to equip participants with accurate information regarding adolescent sexual health development, communication skills and confidence to support adolescents in making healthy sexual decisions. Two levels of training will be provided with each segment lasting approximately 3 h.

### Sexuality 360@school – social worker and guidance counsellor intervention

The social worker and guidance counsellor intervention aims to equip participants with accurate information regarding sexual health community resources and critical case management skills to support adolescents in sexual health crisis. School social workers or guidance counsellors are usually the first point of contact for students regarding sexual issues and for obtaining referrals to relevant sexual health services or counselling supports. Hence, it is essential to equip this staff with sex-positive attitudes and comprehensive knowledge of sexual health services in the community. Two levels of training will be provided with each segment lasting approximately 1.5 h.

### Sexuality 360@school – parent intervention

Parents are the primary sexuality educators for their children and should be equipped and encouraged to leverage teachable moments to talk about sexual health at home. Each school will have two school-based parent trainings to teach skills for discussing sexuality, relationships and growing up. In addition, informational letters explaining topics covered in student workshops with special tips for parents will be shared with parents through the school. Two levels of training will be provided with each segment lasting approximately 2 h.

### Aims

The aim of this study is to evaluate the effectiveness of *Sexuality 360@School*. The program’s key objective is to empower adolescents and key adults in their support networks with the sexual health knowledge, attitudes, skills and connection to motivate healthy sexual decision-making. The ultimate goal of the program is to equip adolescents to engage in safer sex practices and delay the initiation of sex.

### Hypotheses

We hypothesise that each of the target audiences: students, teachers and school management, social workers and guidance counsellors, and parents will increase in knowledge, understanding, awareness, attitudes, self-efficacy and confidence to support healthy sexual decisions among students over the course of the program.

## Methods

### Setting and design

The proposed study is a prospective longitudinal study design implemented in six secondary schools in Hong Kong over two academic years.

### Participants

Recruitment is under way now. The program has successfully recruited six schools to participate, double the number originally anticipated due to strong interest from secondary schools who have a history of working with Mother’s Choice (the non-profit implementing the program).

Since the workshops take place during school hours, all students are required to attend. Parents of students may choose to opt-out of the workshops’ evaluation process. Schools send the opt-out consent form to all parents of students who will participate in the intervention at the beginning of the school year. Each enrolled student will participate in a total of six workshops, three workshops per school year. A target of approximately 1200 students will be recruited into the study. Assuming the effect size of the intervention for each form is small (Cohen’s D effect size of 0.2), it is estimated that a sample size of 265 students from each form will be needed to achieve 90% power in detecting a mean difference via paired t-test at a 0.05 significance level (two-sided). Assuming an estimated 10% attrition rate, 294 students are necessary for each form. As there will be four forms participating in the intervention, the total required sample size will be at least 1176 students.

All teachers and school management, social workers and guidance counsellors, and parents of students from the participating schools will be invited to participate in their respective trainings. Participation is voluntary and each participant will be asked to provide consent before joining the intervention.

### Study outcomes

All outcomes will be collected using pre and post surveys with four measurements over the 2-year research period. Each participant will create an anonymous “pseudo-code” for matching pre and post surveys. Student measurement will be conducted at 4 time points as follows: Year 1 baseline – before first workshop (T0) and after the third workshop (T1); Year 2 baseline – before first workshop (T2) and after the third workshop (T3). Measurement for the other components (i.e. teachers/principals, social workers/school counsellors; parents) will also be conducted at 4 points as follows: Year 1 baseline – before level 1 training (T0) and after level 1 training (T1); Year 2 baseline – before level 2 training (T2) and after level 2 training (T3).

The primary outcomes for the student component include:

- Percent increase in students with correct answers on sexual health knowledge questions
- Percent increase in student awareness of values/attitudes motivating healthy sexual decisions
- Percent increase in student understanding and self-efficacy for healthy sexual communication
- Percent increase in students’ intended condom use

The primary outcomes for the teacher and school management component include:

- Percent increase in mean scores in post survey on sexual health knowledge questions
- Percent who feel confident facilitating sexual decision discussions with students after training
- Percent who find the training helpful

The primary outcomes for the school social worker and guidance counsellor component include:

- Percent with increased understanding of Hong Kong sexual health community resources after training
- Percent who feel confident managing adolescents’ sexual health at-risk cases after training
- Percent who find the training helpful

The primary outcomes for the parent component include:

- Percent increase in self-efficacy mean scores on engaging their children in sexual health discussions after the trainings

The survey questions were developed by Mother’s Choice’s sexual health and public health team, modifying existing instruments for the Hong Kong context. The questions have been piloted in Mother’s Choice programs over the past 2 years and have been well received by students. Questions were adapted from existing validated scales including the “Sexual Communications Self-Efficacy Scale” and a puberty questionnaire for the student survey [28]. Evaluation questions from *Get Real* were adapted for our parent program survey [29]. The face validity of the survey items were evaluated by a team of experts including two public health experts and one registered nurse specialized in sexual health.

Students’ sociodemographic characteristics (e.g. gender, cohabiting family member and parents’ education level) along with their views on sexual behaviours will be collected at T0 and T2. All participants will be asked whether they find the intervention information useful and are satisfied with the intervention at T1 and T3.

### Data analysis

Descriptive statistics including means, standard deviations, frequencies and percentages will be used to describe participant socio-demographic information and study outcomes for each form at each time point. The percentage change in students and other study participants correctly answering each sexual health knowledge question and other items will be calculated at the end of year 1 and year 2, using the pre-test as baseline. Paired t-tests will be used to compare the mean change in study outcomes by each form separately at the end of year 1 and year 2, using the pre-test as baseline. Finally, generalized linear mixed-effects models will be used to obtain adjusted estimates.

## Discussion

Lacking any mandatory school-based sexuality education program amidst rising public health concerns related to sexual health, Hong Kong faces an urgent need for a holistic, replicable and sustainable sexual education model. To the best of our knowledge, this is the first comprehensive sexuality education program developed specifically for the Hong Kong context taking a multifaceted approach to develop an ecosystem of sex-positive support within schools. Ultimately, this program aims to not only empower adolescents and their trusted adults in building a supportive environment for sexual health promotion but also construct a learning network to generate longitudinal evidence for the effectiveness of comprehensive sexuality education in improving sexual health outcomes. The program has the potential for expansion through widespread adoption in Hong Kong schools to benefit more adolescents, and to reduce the medical and societal burdens related to crisis pregnancy, sexually transmitted infections and sexual abuse. It could be further circulated throughout other Chinese-speaking contexts to bring a more comprehensive, evidence-based approach to sexual education.

### Current stage of research

Funding for this project began July 25th 2019. The first year of trainings was conducted beginning in September 2019 through summer 2020 with delays due to Covid-19. The second year of recruitment and training is currently underway at time of submission and is expected to be completed by July 2021. Analysis and reporting will take place in mid-to-late 2021.

## Data Availability

The (de-identified) datasets generated and analysed during the current study are available from the corresponding author on reasonable request.

## Abbreviations

HPS: Health promoting school
STI: Sexually transmitted infection
T0, T1, T2, T3: Year 1 baseline – before first workshop (T0) and after the third workshop (T1); Year 2 baseline – before first workshop (T2) and after the third workshop (T3)

## Footnotes

1 According to the 2016 government census data, there were around 376,500 females between the ages of 15 and 24 who have never been married. The live birth rate per 1000 women aged 15–19 and 20–24 years were 2.7 and 17.8 respectively. 4235 live births were reported in these two age groups last year.

2 An estimated 9.5% of the 5123 cases of medical abortion reported by the HA were among women aged 15–24 years. Additionally the Family Planning Association reports 2010 cases of pregnancy termination in 2016.
